# Variable-frequency ultrasound synergistic hot air drying of Cherry: Effect on drying characteristics and physicochemical quality

**DOI:** 10.1016/j.ultsonch.2025.107387

**Published:** 2025-05-18

**Authors:** Hongyang Lu, Fangxin Wan, Zepeng Zang, Yanrui Xu, Kaikai Zhang, Wenli Ma, Xiaopeng Huang, Guojun Ma

**Affiliations:** College of Mechanical and Electrical Engineering, Gansu Agricultural University, Lanzhou 730070, China

**Keywords:** Cherry, VFU, Synergistic hot air drying, Drying characteristics, Physicochemical quality

## Abstract

This study selected (180 min, 300 min and 60 min, 180 min) as the frequency conversion time nodes and set two modes of sequential frequency conversion (25-28-40 kHz) and reverse frequency conversion (40-28-25 kHz). The objective was to investigate the effects of variable-frequency ultrasound synergistic hot air drying (VFU-HAD) on the drying characteristics and physicochemical properties of cherry. The results showed that compared to hot air drying (HAD), variable-frequency ultrasound (VFU) could reduce drying time (26.32–31.85 %) and specific energy consumption (1.07–21.88 %). Furthermore, VFU demonstrated superior efficacy in improving cherry quality. In particular, in the sequential variable frequency mode, this treatment better preserved anthocyanins, total phenolic content (TPC) and citric acid content in cherries, while significantly improving their free radical scavenging ability (DPPH and ABTS), (p < 0.05). Under the 40 kHz (0 min)-28 kHz (180 min)-25 kHz (300 min) mode, cherries exhibited relatively higher overall contents of glucose (588.85 mg/g), fructose (394.15 mg/g), sucrose (26.48 mg/g), and quinic acid (28.37 mg/g). Furthermore, VFU effectively preserved the color of cherries and significantly improved their textural properties, rendering them softer and more elastic. The microscopic structure indicated that VFU drying promoted the formation of micropores on the cherry surface, facilitating uniform moisture migration. Using the Rank-Sum Ratio comprehensive evaluation method, it was determined that under the 40 kHz (0 min)-28 kHz (180 min)-25 kHz (300 min) mode, the RSR score was the highest (0.8097), indicating the best overall quality of dried cherries. The findings were of great significance for the development of fruit and vegetable drying and the improvement of product quality.

## 1 Introduction

Cherries (*Prunus* species, *Rosaceae* family) originated in Europe and Western Asia and have since been widely cultivated worldwide, with major production areas including Turkey, the United States, Chile, and China [1]. Cherries are rich in a variety of bioactive compounds, including vitamins, TPC, total flavonoids, anthocyanins, organic acids, sugars, and alkaloids. These bioactive compounds exhibit multiple health-promoting properties, including anti-inflammatory effects, cancer risk reduction, neuroprotection, and anti-aging benefits, highlighting their significant research and development potential [2,3]. However, due to the high moisture content (80–86 %) of cherries, they were susceptible to microbial infection and rapid spoilage after harvest, with a shelf life of only 7 to 14 days under conventional conditions [4]. How to prolong the product lifespan of cherries and improve their economic value became the core issue in post-harvest processing. Drying, as a commonly used method for storage and processing of fruits and vegetables, inhibited microbial proliferation by reducing the moisture content, thereby extending the storage period and improving the overall utilization of fruits and vegetables [5]. HAD was the most widely used method for drying fruit and vegetables due to its ease of use, low cost and high flexibility. However, HAD had many challenges in drying efficiency and maintaining the quality of cherries. For example, long drying time, high energy consumption, uneven drying, poor color and loss of flavor and nutrients [6]. Therefore, it was important to develop a combined drying technology based on HAD that is highly efficient and better preserves the quality of the cherries.

Ultrasound (US) is a mechanical wave with a frequency of >2 × 10^4^ Hz, and the cavitation, mechanical and thermal effects induced by it during propagation can effectively reduce the mass transfer resistance at the solid–liquid interface, enhance liquid turbulence, and improve the diffusion of water, which can significantly enhance the heat and mass transfer efficiency [[7], [8], [9], [10]]. US, as a dynamic action, can achieve efficient heat exchange of materials, thus achieving the drying purpose [11]. Existing studies have demonstrated that US offers distinct advantages in enhancing heat and mass transfer efficiency while improving product quality. Romero et al. [12] applied US technology to blackberry drying and obtained a product with improved functional properties. Tan et al. [13] demonstrated that ultrasound-assisted hot air drying of sea buckthorn not only reduced drying time but also enhanced the retention rates of total flavonoids and total phenolic compounds. In their study of US synergistic hot air drying of cherries, Lu et al. [14] found that ultrasonic treatment significantly affected the retention of nutritional components. Through numerical investigations of ultrasonic enhanced convective heat transfer in liquids, Cai et al. [15] demonstrated that US can significantly increase the heat transfer coefficient at liquid surfaces.

However, the traditional ultrasonic drying generally acts on the material at a fixed frequency, and although there is a certain effect in the drying process, there are still issues including weak cavitation and suboptimal ultrasonic energy utilization [16]. Studies have shown that the US frequency has an impact on the drying effect, where low-frequency US penetrates effectively, reducing the material's boundary layer thickness and promoting water evaporation, and high-frequency ultrasonic energy can be effectively transmitted to enhance the diffusion of water within the material. Therefore, the use of VFU can better combine the advantages of low-frequency and high-frequency US to address the requirements of different drying stages. Xu et al. found that VFU in sequential variable-frequency mode (25-28-40 kHz) significantly increased rose polyphenol content [17]. In addition, Xu et al. found that VFU drying provides a solution to the uneven energy distribution and directional sensitivity associated with CFU [18]. Jin et al. analyzed the role of ultrasonic pretreatment in the sequential variable-frequency mode (20–40 kHz) on corn gluten meal and found that the method had a positive effect on peptide preparation [19]. However, at present, VFU drying is less applied in fruit and vegetable processing, and exploring whether it can enhance the drying characteristics and quality of fruit and vegetables is an issue of concern.

This study investigated the effects of VFU-HAD technology on the drying characteristics and physicochemical quality of cherries. By applying VFU technology, different ultrasonic frequencies were utilized at different stages of the cherry HAD process to optimize the drying process, increase drying efficiency and ameliorate the flavor, appearance and nutritional quality of dried cherries.

## 2 Materials and methods

### 2.1 Experimental materials

Fresh cherries (Meizao) used in the experiments were obtained from a cherry plantation area in Tianshui City, Gansu Province, with a wet-base moisture content of 82.9 ± 0.5 %, and were refrigerated in a laboratory constant-temperature and humidity chamber (2–4 °C) immediately after picking. To ensure experimental accuracy, cherries of uniform size and without damage were used as test materials before each trial and stabilized at room temperature (25 ± 0.2 ℃) for 1.5 h.

### 2.2 VFU-HAD cherry

**Fig. 1:**
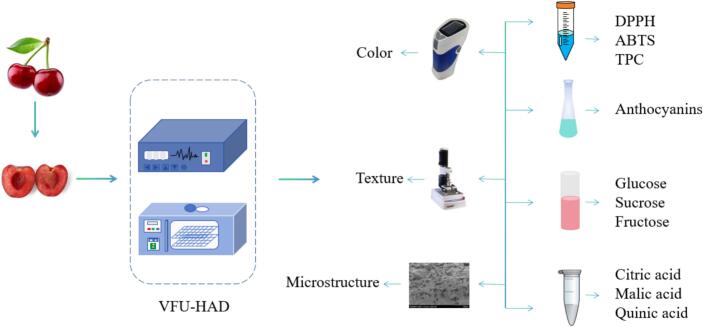
Experimental procedure diagram for VFU-HAD of cherries.

The experimental procedure for VFU-HAD of cherries is displayed in Fig. 1:

This experiment chiefly aimed at exploring the effects of CFU and VFU combined with HAD on the drying characteristics and quality of cherries. CFU drying tests were performed at 25 kHz, 28 kHz, and 40 kHz. Based on the CFU experimental results, the baseline drying parameters were established: hot air temperature at 60°C and US power at 60 W. Meanwhile, cherry shrinkage and color changes during drying served as the basis for determining frequency conversion time nodes and developing the VFU drying experimental protocol.

Cherries (200 ± 0.5 g) were immersed in a solution containing 2 % Na_2_CO_3_ and 1 % ethyl oleate for 10 min to remove the waxy surface layer. After draining surface moisture, the fruits were longitudinally sliced using a vegetable slicer to remove pits. Prepared samples were uniformly arranged on drying trays with cut surfaces facing upward and loaded into a preheated electric hot-air circulating oven (YQ101-0A-4A, Beijing Yu Qin Teng Da Pharmaceutical Equipment Co., Ltd., China). Weight measurements were recorded every 30 min using an electronic balance (AUY220, Shimadzu Corporation, Japan), with each measurement completed within 15 s, until reaching 15 % moisture content [14].

Color and appearance quality play an important role in the production and processing of fruits, vegetables, and food, as they are key indicators of quality changes. However, color, as a crucial sensory attribute, means that vivid and stable coloration attracts consumers [20,21]. In addition, the shrinkage phenomenon generated during the drying process will cause product surface cracking, rehydration ability reduction, while affecting the drying rate and heat and mass transfer efficiency, indirectly affecting the texture quality and taste of the dried product [22,23]. Table 1, Table 2, Table 3 show the shrinkage and color changes of cherries at different time points during drying under three CFU conditions. By comparing the cherry samples at different frequencies in Table 1, Table 2, Table 3, it was found that between 0–180 min, cherry shrinkage was lowest at 25 kHz, in the range of 180–300 min it was lowest at 28 kHz, and after more than 300 min, it was lowest at 40 kHz. These results are consistent with the data presented by Ying et al. on segmented VFU-HAD of rhubarb [24]. From the color perspective, between 0 and 60 min, cherries dried under 40 kHz conditions exhibited the smallest change in total color difference (ΔE). Between 60 and 180 min, the smallest ΔE variation was observed under 25 kHz conditions, while after 180 min, the ΔE value under 28 kHz conditions was lower than that of samples dried under 25 kHz and 40 kHz conditions. Considering all factors, 180 min, 300 min and 60 min, 180 min were chosen as the frequency conversion time nodes. The variable-frequency modes are classified into two types, from low frequency to high frequency (sequential variable-frequency ultrasound, SVU) and high frequency to low frequency (reverse variable-frequency ultrasound, RVU) [24]. The specific scheme is shown in Table 4.

**Table 1:** Shrinkage and colors changes of cherries under 25 kHz conditions.

25 kHz									
Time	0 min	60 min	120 min	180 min	240 min	300 min	360 min	420 min	480 min
Shrinkage	0	10.35 %	25.58 %	45.68 %	68.94 %	75.64 %	78.20 %	80.02 %	81.49 %
ΔE	0	4.71	6.12	10.22	12.72	13.66	14.51	14.64	15.51

**Table 2:** Shrinkage and colors changes of cherries under 28 kHz conditions.

28 kHz								
Time	0 min	60 min	120 min	180 min	240 min	300 min	360 min	420 min
Shrinkage	0	11.28 %	33.26 %	51.17 %	61.85 %	73.08 %	80.20 %	84.76 %
ΔE	0	3.07	7.05	10.08	10.28	11.69	12.83	13.32

**Table 3:** Shrinkage and colors changes of cherries under 40 kHz conditions.

40 kHz							
Time	0 min	60 min	120 min	180 min	240 min	300 min	360 min
Shrinkage	0	17.13 %	38.84 %	55.86 %	73.70 %	78.59 %	82.06 %
ΔE	0	2.17	8.15	10.48	11.08	11.35	11.56

**Table 4:** Experimental scheme of VFU-HAD cherry.

Program name	Time nodes of frequency conversion	Variable-frequency schemes
SVU1	180 min, 300 min	25 kHz (0 min)-28 kHz (180 min) −40 kHz (300 min)
RVU1	180 min, 300 min	40 kHz (0 min)-28 kHz (180 min) −25 kHz (300 min)
SVU2	60 min, 180 min	25 kHz (0 min)-28 kHz (60 min)-40 kHz (180 min)
RVU2	60 min, 180 min	40 kHz (0 min)-28 kHz (60 min)-25 kHz (180 min)

### 2.3 Moisture ratio and drying rate

Cherry moisture ratio and drying rate were calculated as shown in (1) and (2).

(1) $$\mathrm{DR}=\frac{M_{t1}-M_{t2}}{t_{2}{-t}_{1}}$$

(2) $$\mathrm{MR}=\frac{M_{t}-M_{e}}{M_{d}-M_{e}}$$

where *M_t_* is the weight of cherries at moment t, g/g; Me is the weight at equilibrium, g/g; *M_d_* is the initial weight of cherries, g/g; *M_t1_*, *M_t2_* are the moisture content at moment t_1_, t_2_, g/g.

### 2.4 Computation of specific energy consumption

Following the method of Jahanbakhshi et al. [25], the specific energy consumption of the ultrasonic generator and the hot air box in the drying process was calculated as shown in (3).

(3) $${{\mathrm{SEC}=SEC}_{\mathrm{hot}}+SEC}_{\mathrm{ult}}$$

where ${\mathrm{SEC}}_{\mathrm{ult}}$ and ${\mathrm{SEC}}_{\mathrm{hot}}$ are the specific energy consumption of the ultrasonic generator and hot air box in the dewatering process respectively.

### 2.5 Color measurement

The color of the cherries was assessed with a colorimeter (CR-410, Konica Minolta, Japan) and the formula was calculated as follows:

(4) $$\Delta E=\sqrt{{\left(L^{*}-L\right)}^{2}+{\left(b^{*}-b\right)}^{2}+{\left(a^{*}-a\right)}^{2}}$$

where Δ*E* is the total color change; L*, a*, b* and *L, a, b* are the luminance, red-green and blue-yellow values for dried and fresh cherries, respectively.

### 2.6 Determination of soluble sugars and organic acids

#### 2.6.1 Preparation of extract

Dried cherries (0.5 g) were crushed with liquid nitrogen and placed in a container with 25 mL (75 %) ethanol solution. The samples were mixed in a thermostatic shaker (THZ-98B, Shanghai Boxun Medical Biological Instrument Co., Ltd., Shanghai, China) for 48 h (25 ℃, 120 r/min, darkness) and centrifuged in a centrifuge (TD5Z, Hunan Duo Heng Instrument and Equipment Co., Ltd., Changsha, China) for 15 min (4 ℃, 5500 r/min). The supernatant was extracted and retained at 4 ℃. This was used for the determination of the physical and chemical quality of cherries.

#### 2.6.2 Analysis of soluble sugars and organic acids content

The soluble sugars and organic acids were detected in dried cherries using an HPLC system (Agilent 1100, Agilent Technology Co., Ltd, USA). Chromatographic conditions:

Soluble sugars: chromatographic column (ZORBAX NH2); mobile phase: acetonitrile-ultrapure water (75:25); column temperature: 30 ℃; flow rate: 1 ml/min; detection wavelength: 260 nm.

Organic acids: chromatographic column (XSelect HSS T3); mobile phase: 0.2 % phosphoric acid solution-methanol 95:5; column temperature: 30 ℃; flow rate: 1 ml/min; detection wavelength: 210 nm.

### 2.7 Extraction and determination of anthocyanins

The methodology described by Taghavi et al. [26] was adapted and used. Precisely weigh 0.5 g of dried cherries, add 20 ml of a mixture of methanol: ultrapure water: concentrated hydrochloric acid analytically pure (80:20:1), grind rapidly in a mortar, and transfer completely to a conical flask. The subsequent steps were the same as those described in Section 2.5.1. The supernatant was collected, and the absorbance values of the extracts were measured at wavelengths 530 nm and 600 nm using a spectrophotometer (N4, Shanghai Yidian Analytical Instrument Co., Ltd., Shanghai, China). The relative anthocyanin content was calculated using Equation (5).

(5) $$Q=({\mathrm{OD}}_{530}-0.25\times {\mathrm{OD}}_{600})/M$$

*Q*: Relative anthocyanin content, DW/g; *OD_530_*: Absorbance value at wavelength 530 nm; _OD600_: Absorbance value at wavelength 600 nm; *M*: Mass of dried cherries, g.

### 2.8 Determination of TPC

The TPC in cherries was quantified using the Folin-Ciocalteu reagent method according to the method of Xu et al. [27].

### 2.9 Determination of antioxidant activity

#### 2.9.1 DPPH free radical scavenging activity

The protocol described by Wang et al. [28] was used with some modifications. 70 μL of the extract was added to 3.0 mL of 10^-4^ mol/L DPPH methanol solution and stored in the dark to oscillate for 30 min. Using anhydrous methanol as a control, the absorbance values at 515 nm were determined as follows.

#### 2.9.2 ABTS radical scavenging activity

Based on the approach described by Zang et al. [29], with some modifications. Ten milliliters of 7 mmol/L ABTS solution was mixed with 440 µL of 140 mmol/L potassium persulfate solution and allowed to stand in the dark at room temperature for 12 to 16 h. The mixed solution was diluted with 0.1 mol/L phosphate buffer (pH 7.4) to obtain an absorbance value of 0.7 ± 0.05, which served as the ABTS stock solution. The extract (30 µL) was mixed with 3 mL of ABTS stock solution, shaken for 5 min, and allowed to react in a 30 ℃ water bath (HH.S21-6, Shanghai Boxun Medical Biological Instrument Co., Ltd., Shanghai, China) for 30 min. The absorbance at 734 nm was measured using 0.1 mol/L phosphate buffer as a control.

### 2.10 Texture

The texture analysis followed the approach described by Lu et al. [14], with minor adjustments. The texture of dried cherries was measured using a texture meter (TA-XT plus, Stable Micro Systems, UK). A P/36 flat-bottomed probe was used to compress the cherry stem by 60 %. The pre-test, mid-test and post-test velocities were 2 mm/s, 0.5 mm/s and 3 mm/s, respectively, with a trigger force of 0.50 N.

### 2.11 Microstructure

Following the protocol of Lu et al. [14], a scanning electron microscope (S3400N, Hitachi, Japan) was used to observe the surface microstructural changes of the cherry samples at 250× magnification.

### 2.12 Rank-Sum Ratio comprehensive evaluation method

To determine the optimal frequency conversion conditions for cherries, the Rank-Sum Ratio was used for comprehensive evaluation of cherry quality indicators. This method transforms ranked indicator values into a dimensionless statistic (RSR) through a ranking evaluation process. The resulting RSR values enable comparative evaluation of different test conditions. This approach is particularly suitable for comprehensive evaluation indicators with different measurement units [30]. The detailed calculation procedure is presented below.

#### 2.12.1 Constructing the matrix

Assuming that the number of cherry drying programs is n and the number of evaluation indicators is m, construct the data matrix (n × m) and note the matrix A.

#### 2.12.2 Rank matrix

Through the matrix A, the rank of each indicator in each program is calculated, where the positive indicators are ranked in positive order and the negative indicators are ranked in inverse order, and the average value is taken if the data are the same. Obtain the rank matrix, notation $R={\left(R_{\mathrm{ij}}\right)}_{m\times n}$.

#### 2.12.3 Calculating Rank-Sum Ratio

When the weights are equal,

(6) $${\mathrm{RSR}}_{i}=\frac{1}{n\times m}\sum_{j=1}^{m}R_{\mathrm{ij}}$$

When the weights are different,

(7) $${\mathrm{WRSR}}_{i}=\frac{1}{n}\sum_{j=1}^{m}{W_{j}R}_{\mathrm{ij}}$$

where *R_ij_* is the rank of the j th indicator for the i th program; *W_j_* is the weight of the j th indicator; The weights sum to 1.

#### 2.12.4 Sorting

The RSR (WRSR) estimates were calculated to obtain a direct or staged ranking of the evaluation subjects. The higher the value of ${\mathrm{RSR}}_{i}$, the more favourable the evaluation object.

### 2.13 Statistical analysis

IBM SPSS Statistics 27 and Origin 2022 software were used to analyze the experimental data. Significance between different samples was determined by Duncan's multiple range test in analysis of variance (ANOVA), with *P < 0.05* indicating a significant difference. Each test was repeated three times, and the mean values were used for statistical analysis.

## 3 Results and discussion

### 3.1 Effect of VFU-HAD on drying characteristics of cherries

**Fig. 2:**
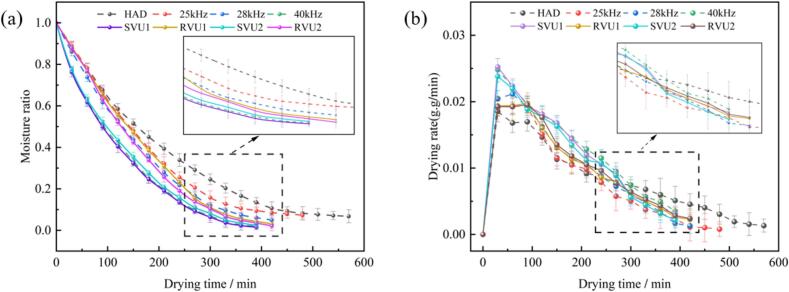
Moisture ratio (a) and drying rate (b) of cherries under different drying conditions.

Fig. 2 shows the moisture ratio versus drying rate curves for cherries under different drying conditions. During CFU drying, higher US frequencies increased the drying rate and reduced drying time. Compared to conventional HAD, VFU-HAD reduced drying time by 26.32–31.58 % and improved drying rate. The drying times of SVU1, RVU1, SVU2, and RVU2 were 390 min, 420 min, 390 min, and 420 min, respectively, in the VFU mode. Compared to the 25 kHz control, drying times for SVU1, RVU1, SVU2, and RVU2 were reduced by 18.75 %, 12.50 %, 18.75 %, and 12.50 %, respectively, which suggests that VFU has a positive impact on reducing the drying time of cherries. VFU generates more cavitation bubbles than CFU, disrupting the cherry surface and enhancing internal moisture diffusion, which breaks the continuity of the cherry surface and promotes the diffusion of internal moisture, thus reducing drying time. Hasanzadeh et al. showed that VFU increases cavitation yield and promotes mass transfer processes [31]. Under the SVU1 and SVU2 modes, the drying rate of cherries between 240 min and 420 min was higher than that of the control group (25 kHz). Thus, VFU improves the drying rate during cherry dehydration. This is because, in the VFU drying process, the cavitation bubbles are initially subjected to a shear force, and under the action of US at different frequencies, they are continuously compressed, thinned and cyclically grown, which strengthens the cavitation effect and promotes the mass transfer effect within the cherries, thus improving the drying rate [17]. Meanwhile, the ultrasonic mechanical effect enlarges the microporous pipeline inside the cherry, increases the turbulence of the internal moisture, reduces the resistance between the inside and outside of the diffusion boundary and improves the mobility of the moisture [32].

### 3.2 Specific energy consumption (SEC)

**Fig. 3:**
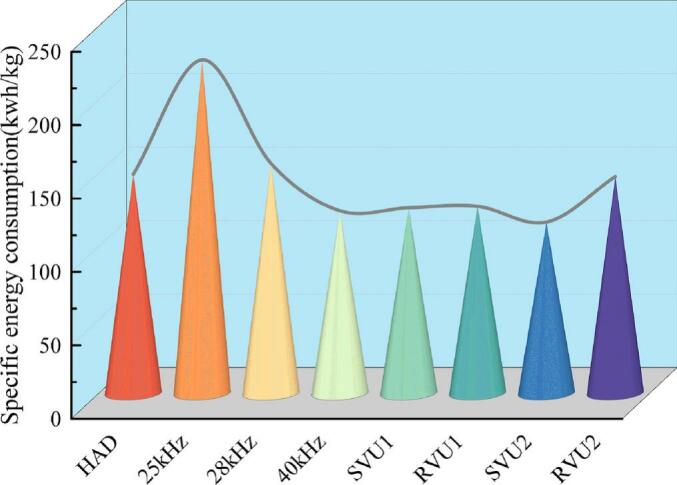
Specific energy consumption during cherry drying under different drying conditions.

Energy consumption critically impacts fruit and vegetable drying processes and has a direct impact on production costs, environmental sustainability and product quality. Technological innovations like combined and segmented drying, an optimal balance among high efficiency, low carbon footprint, and superior quality can be achieved. Fig. 3 shows the SEC of cherries under different drying conditions. The SEC was reduced by 15.29 %, 14.65 %, 21.88 % and 1.07 %; 44.37 %, 43.95 %, 48.70 % and 35.05 % in SVU1, RVU1, SVU2 and RVU2 modes respectively compared to the control group (hot and 25 kHz). VFU significantly reduced SEC and enhanced drying efficiency. The 40 kHz and variable-frequency modes yielded lower SEC, with SVU2 achieving the minimum (116.41 kWh/kg). This reduction stems from shorter dehydration times and accelerated moisture migration. In summary, VFU demonstrates significant benefits and potential for energy savings, providing valuable insights for industrial applications.

### 3.3 Anthocyanins and color

**Fig. 4:**
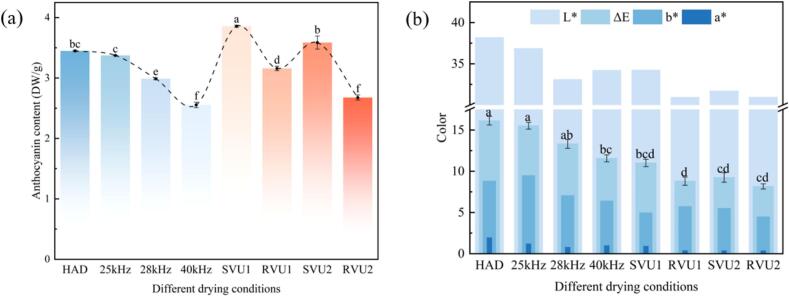
Anthocyanin content and color variation of cherries under different drying conditions.

Anthocyanins, natural water-soluble pigments abundant in fruits and vegetables. Elevated anthocyanin content imparts cherries with deep red or purple color, enhancing consumer appeal [33]. Fig. 4(a) and (b) show the changing patterns of cherry anthocyanin content and color under different drying conditions. Under CFU drying, the anthocyanin content showed a decreasing trend as the frequency increased from 25 kHz to 40 kHz. This phenomenon occurs because anthocyanins are heat sensitive compounds. Higher US frequencies intensify cavitation, generating localized high temperatures via asymmetric bubble collapse and microjets, ultimately inducing thermal degradation of the anthocyanins [34]. Meanwhile, water molecules produce hydroxyl radicals under cavitation, which cleave the benzene ring on anthocyanins, also contributing to anthocyanin degradation [35]. In contrast, the anthocyanin content was 3.86 DW/g and 3.59 DW/g in SVU1 and SVU2 modes, respectively. The anthocyanin content increased by 14.30 % and 6.30 % respectively relative to the control (25 kHz). This suggests that VFU can better retain anthocyanins and effectively inhibit anthocyanin degradation. This may be due to the fact that VFU effectively preserves anthocyanins by dynamically adjusting the frequency to make the size and distribution of the cavitation bubbles more uniform, avoiding the high intensity mechanical and thermal effects at a fixed frequency. Furthermore, under VFU drying, the value of color difference of cherries gradually decreased with increasing frequency. This may be due to the fact that higher frequency US promotes moisture mobility, intensifies the mass transfer process and shortens the drying time, thus reducing the Maillard reaction and bringing the color of the dried samples closer to that of the fresh samples [36]. Cherry color change negatively correlates with anthocyanin retention [37]. The ΔE values of the cherry samples after VFU drying were all lower than those of the controls (25, 28 or 40 kHz). In particular, the apparent quality of dried cherries was more pronounced in the RVU1 mode, with L* (30.94), a* (0.16), b* (4.47) and ΔE (8.16) lower than in the controls (25, 28 or 40 kHz). Collectively, VFU protects cherry color during dehydration, reducing browning and pigment degradation and enhancing post-drying quality.

### 3.4 Soluble sugars and organic acids

**Fig. 5:**
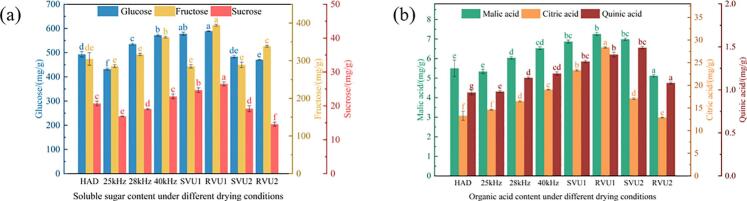
Soluble sugars and organic acids content of cherries under different drying conditions.

Fig. 5 shows the changes in the content of glucose, fructose, sucrose, malic acid, citric acid and quinic acid in dried cherries under different drying conditions. Under the RVU1 mode, the highest contents of glucose (588.85 ± 1.37 mg/g), fructose (394.15 ± 2.30 mg/g), sucrose (26.48 ± 0.59 mg/g) and quinic acid (28.37 ± 0.14 mg/g) were found in the cherry samples, which were 1.36, 1.38, 1.57 and 1.33 times higher, respectively. This may be due to the fact that the high frequency US created more micropores during the first drying stage, facilitating the release of monosaccharides and organic acids. In the second and third phases, US at lower frequencies has greater penetrating power and generates greater shear and mechanical forces, causing the cells to oscillate and rupture, releasing more sugars and acidic compounds [38]. In comparison, the RVU2 mode showed lower concentrations of sucrose, malic acid, and quinic acid. This may be attributed to the prolonged low frequency US action, which increases the time for degradation reactions to occur in sugars and organic acids. Tao et al. [39] obtained comparable results. Dried cherries are high in glucose and fructose and low in sucrose and organic acids. This is due to the irreversible conversion of sucrose to glucose and fructose by the action of converting enzymes, while sugars and organic acids have some antagonistic effects [29].

### 3.5 TPC and antioxidant capacity

**Fig. 6:**
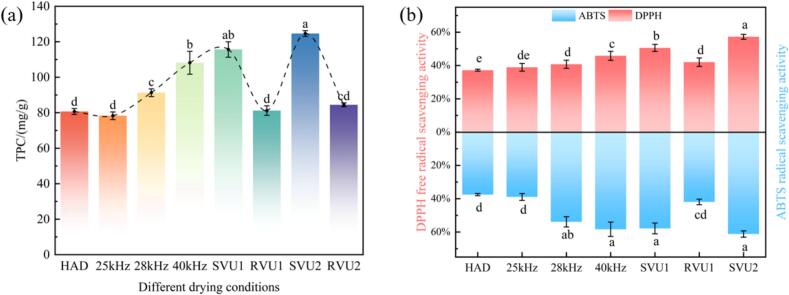
TPC, antioxidant activity (DPPH, ABTS) of cherries under different drying conditions.

Antioxidant activity is a key indicator of fruit and vegetable nutrition, which can be used to slow ageing and prevent chronic diseases by scavenging or inhibiting free radicals and reducing cellular damage caused by oxidative stress. Research has indicated that the antioxidants found in cherries are mainly composed of TPC and anthocyanins. The TPC and antioxidant capacity of cherries under different drying conditions are illustrated in Fig. 6(a) and (b). As opposed to the control group (25 kHz), the TPC of cherries increased by 47.67 %, 3.70 %, 59.13 %, and 7.84 % under the SVU1, RVU1, SVU2, and RVU2 modes, respectively. Meanwhile, DPPH and ABTS increased by 29.91 %, 7.86 %, 47.03 %, 5.03 % and 48.56 %, 7.72 %, 57.21 %, 25.22 %, respectively. In addition, the TPC (124.62 mg/g) and the antioxidant capacity (DPPH: 57.25 % and ABTS: 61.18 %) of the cherries were more pronounced in the SVU2 mode. This suggests that VFU has a beneficial effect on the TPC and antioxidant capacity of cherries. This may be due to the fact that the shear force generated by variable frequency US enhances the disruption of intermolecular chemical bonds, hydrolyzing phenolic acids to phenolic derivatives and increasing the output of phenolic compounds [40]. At the same time, US disrupts the structure of the enzyme through cavitation shear, inhibits the activity of polyphenol oxidase and reduces the enzymatic reaction rate, thus reducing the degradation of phenolics [41,42]. Compared to the other two inverter modes, RVU1 and RVU2 modes exhibited lower DPPH and ABTS radical scavenging activities in cherries. This has been attributed to the fact that VFU can produce stronger transient cavitation than CFU, and the subsequent collapse of unstable bubbles generates free radicals that contribute to the degradation of phenolic compounds and other antioxidants, thus reducing antioxidant capacity [43]. Overall, the antioxidant capacity and TPC of cherries showed similar trends, indicating a strong correlation between TPC and antioxidant activity [44].

### 3.6 Texture and microstructure

**Fig. 7:**
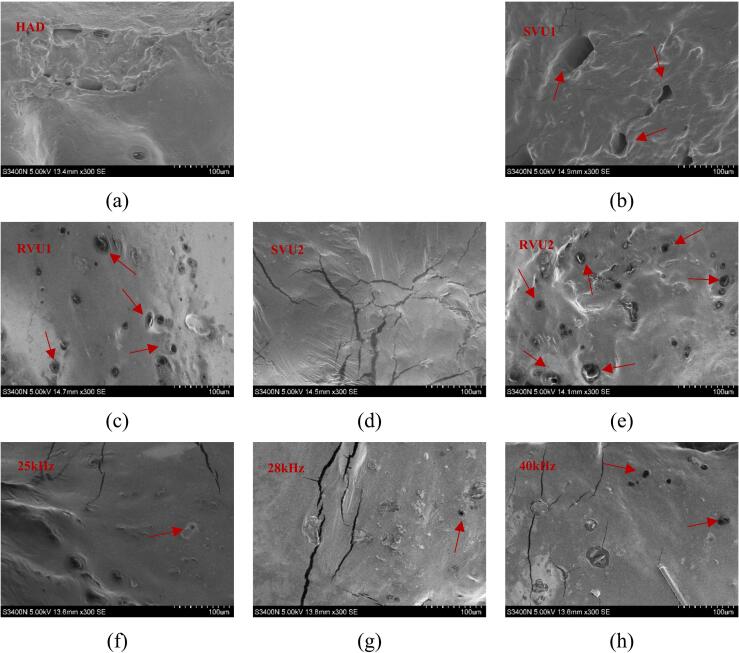
Surface microstructure of cherries under different drying conditions.

Texture is a crucial quality attribute of fruit and vegetables. For cherries, it is a key organoleptic quality indicator. It also has a major impact on consumer acceptance. Microstructural changes are key determinants of cherry internal moisture migration, heat and mass transfer during drying. However, the microstructure of the cherry also affects its texture to some extent. The textural properties of cherries can be characterized by hardness, springiness, cohesiveness, gumminess, chewiness, and resilience (Table 5), and the microstructure of dried cherries is shown in Fig. 7. VFU drying reduced hardness, gumminess, chewiness and improved springiness and resilience of dried cherries compared to the control (25 kHz), but the effect on cohesiveness was not significant. The best cohesion (0.75 ± 0.02) and elasticity (81.66 % ± 2.62) were observed in the SVU1 mode. This phenomenon may originate from carboxyl groups forming hydrogen bonds with hydroxyl groups in pectin molecules, creating a flexible and elastic hydrogen-bonded network structure that enhances the elasticity of dried cherries [45]. Concurrently, the mechanical effects of US induce scission of polysaccharide chains, resulting in reduced molecular weight. This depolymerization subsequently alters the rheological properties within cherry tissues, manifesting as decreased system viscosity and ultimately leading to diminished chewiness in dried cherries [46]. Under alternating ultrasonic frequencies, shear forces and transient cavitation created extensive microcracks and channels. These structural changes loosened the tissue framework, enhancing moisture transport and ultimately decreasing product hardness, as evidenced in Fig. 7(b) and (d) [47,48]. Observation of Fig. 7(c) and (e) shows that under RVU1 and RVU2 modes, the surface of the cherry stem produces many micropores and the surface is relatively flat, which suffers relatively little damage. Azam et al. also found that lettuce leaves suffered less mechanical damage during variable frequency drying [49].

**Table 5:** Texture of dried cherries under different drying conditions.

Drying conditions	Hardness (N)	Springiness (%)	Cohesiveness	Gumminess (N)	Chewiness (N)	Resilience (%)
HAD	14.98 ± 0.1^c^	80.81 ± 2.76^cd^	0.65 ± 0.03^cd^	9.71 ± 0.58^b^	7.87 ± 0.77^ab^	32.08 ± 0.01^bc^
25 kHz	15.31 ± 0.18^b^	73.21 ± 0.05^bc^	0.63 ± 0.01^de^	9.66 ± 0.24^b^	6.39 ± 0.52^abc^	26.81 ± 0.03^ab^
28 kHz	13.62 ± 0.01^d^	76.36 ± 0.05^a^	0.71 ± 0.01^ab^	9.02 ± 0.79^bc^	7.15 ± 1.12^abc^	28.40 ± 0.05^a^
40 kHz	17.82 ± 0.10^a^	75.87 ± 0.05^ab^	0.68 ± 0.01^bc^	12.51 ± 0.16^a^	9.27 ± 0.80^a^	30.87 ± 0.02^bc^
SVU1	8.36 ± 0.05^h^	81.66 ± 2.62^ab^	0.75 ± 0.02^a^	5.80 ± 0.16^d^	4.74 ± 0.21^cd^	30.74 ± 0.02^cd^
RVU1	12.77 ± 0.14^e^	73.68 ± 3.22^d^	0.60 ± 0.01^e^	8.34 ± 0.82^bc^	6.20 ± 1.16^bcd^	31.60 ± 0.05^de^
SVU2	11.44 ± 0.01^f^	75.03 ± 3.64^c^	0.68 ± 0.01^bc^	7.74 ± 0.10^c^	5.81 ± 0.37^bcd^	29.47 ± 0.02^cd^
RVU2	8.91 ± 0.04^g^	74.91 ± 2.99^bc^	0.64 ± 0.01^cde^	5.73 ± 0.04^d^	4.29 ± 0.12^d^	27.65 ± 0.03^e^

### 3.7 Cluster analysis (CA)

**Fig. 8:**
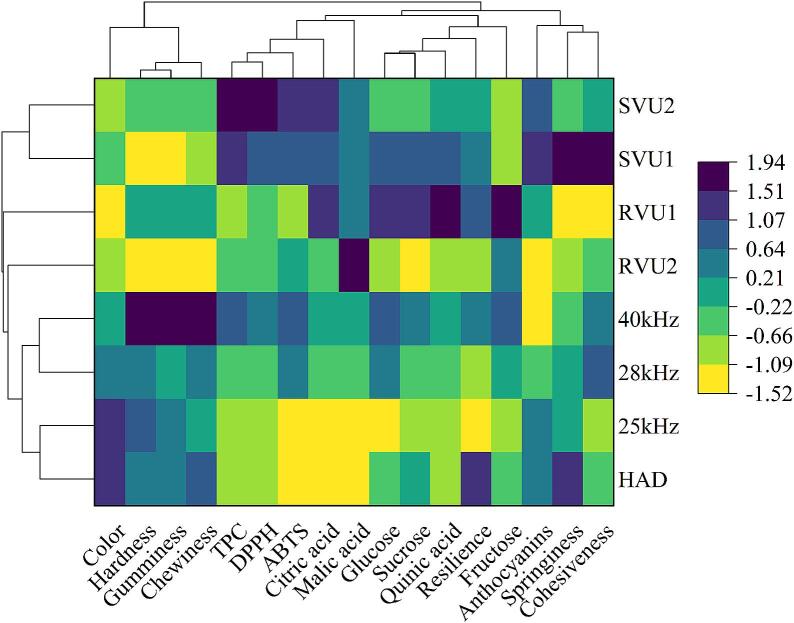
Clustered heat map of cherry quality characteristics under different drying conditions.

Fig. 8 shows the clustered heat map of cherry quality characteristics under different drying conditions. Overall, samples of SVU and RVU types (SVU1, RVU1, SVU2, RVU2, and HAD) show significant differences in cherry quality characteristics compared to samples of other frequency types (25 kHz, 28 kHz and 40 kHz). The 40 kHz samples exhibited the highest and most similar values for hardness, gumminess, and chewiness. The SVU2 samples had higher levels of TPC, DPPH, ABTS, and citric acid. The SVU1 samples showed greater values for anthocyanins, springiness, and cohesiveness. Meanwhile, the RVU1 samples had higher and similar values for glucose, sucrose, quinic acid, citric acid, fructose, and resilience. The RVU1 samples were more clearly clustered than the 25 kHz samples, with the greatest differences in cherry quality characteristics.

### 3.8 Rank-sum ratio evaluation

Color, TPC, anthocyanins, DPPH, ABTS, glucose, fructose, sucrose, malic acid, citric acid, and quinic acid were used as evaluation indicators. In accordance with the Rank-Sum Ratio comprehensive assessment standard (Table 6), the indicators were ranked using the Rank-Sum Ratio method. The overall quality of the cherries improves with higher RSR values or grades (Table 8) [50]. The weights of each indicator of Cherry were obtained by entropy weighting method, as shown in Table 7. Among them, Fructose ($w_{j}=13.922$), DPPH ($w_{j}=10.109$), TPC ($w_{j}=12.776$), Quinic acid ($w_{j}=11.608$), ABTS ($w_{j}=8.309$) had higher weights. This indicates that these indicators have an important effect on the quality of the cherries, which also shows that drying conditions have a notable effect on the bioactive compound content in cherries. The results of the overall Rank-Sum Ratio evaluation showed that the RVU1 condition had a better overall quality of dried cherries with the highest RSR (0.8097), which was the most desirable drying condition.

**Table 6:** Ranking criteria for comprehensive evaluation of Rank and Ratio.

Grade classification	1	2	3	4	5
RSR threshold	<0.1039	0.1039–0.3351	0.3351–0.5664	0.5664–0.7976	>0.7976

**Table 7:** Weights of the indicators for cherries under different drying conditions.

Indicator names	$e_{j}$	d	$w_{j}\left(\%\right)$
TPC	0.752	0.248	12.776
DPPH	0.805	0.195	10.109
ABTS	0.839	0.161	8.309
Anthocyanins	0.868	0.132	6.773
Glucose	0.882	0.118	6.072
Sucrose	0.883	0.117	6.038
Citric acid	0.817	0.183	9.398
Fructose	0.730	0.270	13.922
Malic acid	0.843	0.157	8.102
Quinic acid	0.775	0.225	11.608
ΔE	0.864	0.136	6.982

**Table 8:** Rank-Sum Ratio comprehensive evaluation results.

Drying conditions	RSR fitted value	RSR Ranking	Grade classification
HAD	0.3208	7	2
25 kHz	0.2291	8	2
28 kHz	0.3893	6	3
40 kHz	0.5121	4	3
SVU1	0.5807	3	4
RVU1	0.8097	1	5
SVU2	0.6724	2	4
RVU2	0.4507	5	3

## 4 Conclusion

To enhance heat and mass transfer efficiency and address the weak cavitation effect and low ultrasonic energy rate in CFU drying, this study combined VFU with HAD to investigate their effects on cherry drying characteristics and physicochemical properties. Results showed that VFU-HAD significantly enhanced moisture migration during drying, reducing drying time (26.32–31.58 %) and SEC (7.14–18.75 %) through alternating ultrasonic frequencies. Meanwhile, the physicochemical properties of the cherries were improved. VFU significantly (*P < 0.05*) improved retention of anthocyanins, TPC, antioxidant properties (DPPH and ABTS radical scavenging), organic acids, and soluble sugars in both SVU1 and RVU1 modes. In RVU1 mode, the color difference of the cherries varied the least and their color was closer to the fresh samples. Compared to CFU, VFU reduced the hardness, chewiness and gumminess of dried cherries and increased their elasticity and softness. The microstructure showed that VFU created more micropores on the surface of the cherries, which promoted uniform evaporation of water and improved drying efficiency. On the basis of cluster analysis and the RSR method, it was concluded that the best overall quality of dried cherries was produced under the RVU1 mode.

In summary, the VFU-HAD technology proved to be highly effective in increasing the drying rate and optimizing the physicochemical properties of cherries. This work can serve as a theoretical and technical reference for the deep processing of cherries.

## CRediT authorship contribution statement

**Hongyang Lu:** Writing – review & editing, Writing – original draft, Visualization, Validation, Software, Methodology, Formal analysis, Data curation, Conceptualization. **Fangxin Wan:** Validation, Resources, Project administration, Funding acquisition. **Zepeng Zang:** Writing – review & editing, Methodology, Investigation. **Yanrui Xu:** Methodology, Investigation. **Kaikai Zhang:** Methodology, Investigation. **Wenli Ma:** Methodology, Investigation. **Xiaopeng Huang:** Validation, Supervision, Resources, Project administration, Funding acquisition, Formal analysis. **Guojun Ma:** Validation, Resources, Methodology.

## Declaration of competing interest

The authors declare that they have no known competing financial interests or personal relationships that could have appeared to influence the work reported in this paper.
